# Editorial: Bioactive natural products for health: isolation, structural elucidation, biological evaluation, structure-activity relationship, and mechanism, volume II

**DOI:** 10.3389/fchem.2026.1884133

**Published:** 2026-06-08

**Authors:** Qin-Ge Ma, Wen-Min Liu, Zhao Geng, Hui-Lian Huang, Chun-Su Yuan, Rong-Rui Wei

**Affiliations:** 1 School of Life Science and Technology and College of Medicine and Health Science, Wuhan Polytechnic University, Wuhan, China; 2 College of Chemistry and Pharmaceutical Engineering, Nanyang Normal University, Nanyang, China; 3 Research and Development Center, Jiangxi Huiren Pharmaceutical Co., Ltd., Nanchang, China; 4 Key Laboratory of Modern Preparation of Traditional Chinese Medicine of Ministry of Education, Jiangxi University of Traditional Chinese Medicine, Nanchang, China; 5 Tang Center for Herbal Medicine Research, Department of Anesthesia and Critical Care, Committee On Clinical Pharmacology and Pharmacogenomics, Pritzker School of Medicine, The University of Chicago, Chicago, IL, United States

**Keywords:** bioactivity, mechanism, medicinal plants, natural products, pharmacology, phytochemistry

Medicinal plants have long been a cornerstone of natural active ingredient discovery, harboring thousands of structurally novel and diverse bioactive products that underpin modern drug development (Guo et al., 2024; Ma et al., 2024a). As a vital pathway for drug discovery, natural products and their derivatives account for a significant proportion of all approved drugs, highlighting their irreplaceable value in pharmaceutical research (Bhawna and Ashwani, 2021). Despite significant progress in isolating natural active products from medicinal plants, numerous species remain underexplored and underdeveloped, with unclear pharmacodynamic material bases, unelucidated structure-activity relationships, and ambiguous mechanisms of action—factors that severely impede the discovery of new plant-derived drugs. Thus, exploring new active molecules from medicinal plants is of profound practical significance for advancing innovative drug development.

Against this backdrop, researchers focus aims to identify new bioactive natural products from medicinal plants using modern extraction and separation technologies (Les et al., 2021; Ma et al., 2026). Aligned with current clinical drug demands, they integrate interdisciplinary approaches—including high-throughput screening, molecular docking, metabolomics, serum pharmacology, network pharmacology, and natural medicinal chemistry—focusing on key areas such as isolation, structural elucidation, biological evaluation, structure-activity relationships, mechanism exploration, and yield improvement of bioactive natural products (Ma et al., 2023; Wei et al., 2026). This research is pivotal to expanding the pool of known bioactive natural products and boosting innovative drug development.

To highlight the latest research status of natural active ingredients in drug research and development, researchers launch this Research Topic, focusing on key research directions: characterization of medicinal plant active ingredients, bioactivity identification of plant extracts, chemistry and biochemistry of plant-derived bioactive natural products, standardization of extracts and natural products (Ma et al., 2024b; Ye, 2019). Manuscripts reporting new medicinal plant-derived compounds with detailed biological activity descriptions are particularly encouraged (Liu et al., 2025; Wei et al., 2025). The researchers anticipate this Research Topic will serve as a platform to accelerate knowledge exchange and drive progress in plant-based bioactive molecule research and innovative drug discovery.

The study by Wang et al. used an integrated approach, combining metabolomic analysis and network pharmacology to unravel salidroside’s role in promoting testosterone secretion in H_2_O_2_-induced TM3 Leydig cells. The findings confirmed that salidroside reduces ROS and MDA levels, increased testosterone, SOD, and GSH levels, ameliorating oxidative stress and protecting Leydig cells. UPLC-QE-Orbitrap-MS identified 28 differential metabolites and key metabolic pathways, including arginine biosynthesis and the TCA cycle, while network pharmacology revealed core targets (AMACR, CYP3A4, etc.), validated by dry-wet analysis. This work is significant for clarifying salidroside’s multi-target, multi-pathway mechanism in promoting testosterone secretion, bridging metabolomic changes with molecular targets. By linking anti-oxidant activity to testosterone regulation, it provides robust scientific evidence for salidroside as a novel strategy for preventing and treating testosterone deficiency. This integrated research design also offers a valuable template for investigating natural compounds’ therapeutic mechanisms, advancing reproductive health research and bringing new hope for patients with testosterone deficiency.

The review by Cao et al. addressed this need comprehensively, systematically profiling both traditional and green extraction technologies for plant extracts and absolutes. It evaluated traditional methods (maceration, percolation, reflux, Soxhlet extraction) and green alternatives (microwave-assisted, ultrasonic-assisted, pressurized liquid, supercritical fluid extraction), comparing their principles, advantages, disadvantages, improvement strategies, and practical applications. Additionally, the review summarized new green extraction solvents, offering multi-perspective insights into their industrial utility. This work is highly valuable for bridging extraction technology research and industrial practice. By providing a holistic comparative analysis, it equips researchers and industry professionals with a clear guide to selecting optimal extraction methods, promoting efficient, sustainable utilization of plant resources. It also highlights the potential of green extraction technologies to address environmental concerns, advancing the development of eco-friendly processes and unlocking new possibilities for plant-derived products across diverse sectors.

The study by He et al. addressed this critical gap through an integrated approach, combining network pharmacology, molecular dynamics simulation, and experimental validation. By screening public databases, the research identified 13 core targets of curcumin in spinal cord injury (SCI), including MMP9, TNF, IL-1*β*, STAT3, and CASP3, and revealed its regulation of HIF pathway, inflammation, and oxidative stress. Notably, molecular docking and dynamics confirmed curcumin’s high affinity with MMP9 (binding energy −8.76 kcal/mol) and stable binding at specific residues, while OGD-induced PC12 cell experiments validated its therapeutic effect and inhibition of MMP9 protein expression. This work is significant for clarifying curcumin’s multi-target, multi-pathway therapeutic mechanism in SCI, highlighting MMP9 as a key mediating target. By bridging *in silico* prediction with *in vitro* validation, it provides robust scientific evidence for curcumin’s clinical potential and lays a foundation for further drug development. This multidimensional research design also offers a valuable template for investigating natural compounds’ therapeutic mechanisms, advancing neurological injury research and bringing new hope for SCI patients.

The study by Eldahshan et al. addressed this gap through a systematic approach, using GC-MS to first characterize the hexane extracts of bitter orange fruits and leaves, and evaluating their anti-oxidant and enzyme inhibitory activities *in vitro*, complemented by *in silico* molecular docking. The findings identified distinct major components in fruit (nootkatone, decyl anthranilate) and leaf (lupeol, linalool) extracts, and confirm their potent inhibitory effects on key enzymes (AChE, BChE, tyrosinase, amylase, glucosidase), with lupeol and acetate derivatives showing optimal binding affinities to target enzymes via docking. This work is significant for valorizing bitter orange waste, highlighting its potential as a sustainable source of antioxidants and functional supplements. By integrating metabolomic profiling with *in vitro* and *in silico* validation, it provides actionable insights for developing natural ingredients for pharmaceuticals, nutraceuticals, and cosmetics. This research also advances our understanding of Citrus bioactivities, paving the way for more efficient utilization of Citrus resources and promoting the development of natural, multi-functional products.

The study by Bory et al. investigated this critical bottleneck by developing fungal library extract conversion and screening in 96-well plate format (FLECS-96), a miniaturized high-throughput platform that streamlined the conversion of fungal strains into chemically characterized extract libraries. Integrating miniaturized liquid culture, simplified sample preparation, systematic metabolomic profiling, and biological evaluation, FLECS-96 overcame the inefficiencies of traditional methods, as validated by its successful rapid identification of compounds active against *S. aureus*. This work represents a significant advancement in antimicrobial discovery, providing a scalable, efficient solution to unlock the potential of fungal resources. By bridging the gap between fungal strain Research Topic and actionable bioactive leads, FLECS-96 accelerates the pace of antimicrobial lead discovery, offering hope for combating drug-resistant pathogens. It also sets a valuable template for high-throughput natural product screening, advancing natural product drug discovery and addressing an urgent unmet clinical need.

The study by Elkhawas et al. filled this gap through a comprehensive approach, combining gas chromatography-mass spectrometry for volatile compound analysis, *in vitro* enzyme inhibition assays (anti-collagenase and anti-elastase), ABTS radical scavenging for anti-oxidant activity, and molecular docking. The findings clearly demonstrated that fresh key lime essential oil exhibited superior anti-aging and anti-oxidant performance: it had lower IC_50_ values for elastase (145.02 μg/mL) and collagenase (63.97 μg/mL) than dry oil, and stronger anti-oxidant activity (37.76 ± 0.80 μM TE/g). Molecular docking further confirmed the strong binding capacity of its main metabolites to target enzymes, supporting its anti-aging mechanism. This work is significant for bridging plant chemistry with cosmetic science, highlighting the impact of fruit processing on bioactive compound retention. By identifying fresh key lime essential oil as a potent natural anti-aging ingredient, it provides valuable insights for cosmetic companies developing natural formulations. It also sets a template for evaluating plant-derived essential oils, advancing the development of safe, effective natural skincare products to meet consumer demand.

Fu et al. used a comprehensive approach, combining *in vitro* experiments on human microvascular endothelial cells (HMEC-1) with network pharmacology, molecular docking, and immunoblotting. The findings confirmed hydroxysafflor yellow A (HSYA) exerted concentration-dependent pro-angiogenic effects without cytotoxicity, identified 121 potential targets, and highlighted key regulatory pathways including HIF-1, PI3K-Akt, and VEGF. Notably, molecular docking and functional validation demonstrated HSYA’s high-affinity binding to HIF-1*α* and MMP9, two hub regulators of angiogenesis, clarifying their critical role in HSYA’s mechanism. This work bridges traditional Chinese medicine (TCM) wisdom with modern pharmacology, providing clear molecular evidence for HSYA’s pro-angiogenic activity. By pinpointing HIF-1*α* and MMP9 as key targets, it lays a foundation for developing HSYA as a novel therapeutic agent for ischemic diseases. This integrated research design also offers a valuable template for investigating bioactive constituents of TCM, advancing the modernization of traditional remedies and unlocking new avenues for treating vascular disorders.

Chen et al. discussed about an integrated phytochemical annotation strategy, combining UPLC-Q Exactive Orbitrap HRMS with an automated workflow that integrated in-house libraries, public databases, and feature-based molecular networking (FBMN). Validated using Xie-Bai-San decoction (XBSD), a classic Traditional herbal formulations (THFs) composed of three herbs, this strategy successfully annotated 218 compounds, including 74 newly reported ones, covering multiple classes such as flavonoids, organic acids, and terpenoids. Notably, it assigned compounds to different confidence levels and identifies 13 constituents unambiguously via reference standards, ensuring reliability and transparency. This work stands out for addressing the key challenges in THF phytochemical analysis: FBMN enables visual clustering of structural analogues, while the integrated workflow enhances efficiency and discriminability of isomers and low-abundance compounds. It not only provides a comprehensive chemical profile of XBSD but also offers a practical framework for rapid, reliable characterization of complex THFs. This strategy bridges the gap between traditional herbal medicine and modern analytical technology, facilitating the standardization and modernization of THFs and laying a foundation for further efficacy and mechanism research.

El-Mordy et al. used an integrated *in vivo* and *in silico* approach, combining network pharmacology, molecular docking, and dynamics with *in vivo* gene expression analysis. By isolating major seed constituents and evaluating their effects, the research demonstrated that *C. olitorius* L. (Molokhia) seed extracts mitigated doxorubicin-induced cardiotoxicity by downregulating pro-inflammatory genes (IL-1*β*, IL-6, TNF-*α*, etc.) and modulating ECG parameters, pinpointing anti-inflammatory action as a key mechanism. This work is notable for bridging traditional knowledge with modern computational and experimental techniques, validating the cardioprotective potential of *Corchorus olitorius* L. seeds while providing actionable mechanistic insights. It paves the way for developing natural, low-toxicity adjuvants to alleviate doxorubicin-related cardiac damage, advancing cardio-oncology research and leveraging plant-based therapies for unmet clinical needs. Further studies are warranted to optimize extraction strategies and validate clinical applicability.
